# Nonoperative treatment for pain sensitization in patients with low back pain: protocol for a systematic review

**DOI:** 10.1186/s13643-022-01927-2

**Published:** 2022-04-04

**Authors:** Tanawin Nopsopon, Areerat Suputtitada, Irin Lertparinyaphorn, Krit Pongpirul

**Affiliations:** 1 Department of Preventive and Social Medicine, Faculty of Medicine, Chulalongkorn University, Bangkok, Thailand; 2 Department of Rehabilitation Medicine, Faculty of Medicine, Chulalongkorn University, and King Chulalongkorn Memorial Hospital, Rama 4 Road, Pathumwan District, Bangkok, 10330 Thailand; 3 Department of Anatomical Pathology, Faculty of Medicine, Chulalongkorn University, Bangkok, Thailand; 4 Department of International Health, Johns Hopkins Bloomberg School of Public Health, Baltimore, MD USA

**Keywords:** Low back pain, Quantitative sensory testing, Nonoperative, Systematic review

## Abstract

**Background:**

Low back pain is a disability that occurs worldwide. It is a heterogeneous disorder that affects patients with dominant nociceptive, neuropathic, and central sensitization pain. An important pathophysiology of low back pain involves pain sensitization. Various nonoperative interventions are available for treatment, but there is inconclusive evidence on the effectiveness of these interventions for pain sensitization, leading to arbitrary nonoperative treatments for low back pain.

**Methods:**

We will conduct a systematic review of RCTs evaluating the effectiveness and safety of nonoperative treatment for pain sensitization in patients with low back pain. The primary outcomes will be static quantitative sensory testing, dynamic quantitative sensory testing, and pain algometry. The secondary outcome will be adverse events. We will search the PubMed, Embase, Scopus, Web of Science, Cumulative Index to Nursing and Allied Health Literature (CINAHL), Cochrane Central Register of Controlled Trials (CENTRAL), and Cochrane Library databases. Two independent authors will screen the titles and abstracts, review full texts, extract data, assess the risk of bias, and evaluate the quality of evidence. We will qualitatively and quantitatively synthesize the results using a random effects model for meta-analysis.

**Discussion:**

This systematic review aims to provide evidence regarding which treatment, if any, provides the greatest benefit for pain sensitization and safety among patients with low back pain. Evidence synthesized from this systematic review will inform clinical practice and further research. Since there is still a small amount of research, additional studies might need to be conducted in the future.

**Systematic review registration:**

Submitted to PROSPERO on March 20, 2021, CRD42021244054

**Supplementary Information:**

The online version contains supplementary material available at 10.1186/s13643-022-01927-2.

## Background

### Description of the condition

Low back pain (LBP) is defined as pain or discomfort localized below the costal margin and above the gluteal crease, with or without referred leg pain [1]. Nonspecific LBP is the most common type of LBP and is defined as LBP without any known specific cause or pathology (e.g., infection, cancer, osteoporosis, inflammation, cauda equina syndrome or fractures) [2]. LBP can be classified into three groups based on the duration of symptoms: acute LBP persists for less than 6 weeks, subacute LBP persists between 6 and 12 weeks, and chronic LBP persists for 12 weeks or more [3, 4].

Low back pain is a major health problem among the global population. There are substantiate numbers of populations suffering from low back pain, with an estimated 33% point prevalence, 65% 1-year prevalence, and 84% lifetime prevalence [5]. LBP is an important cause of global disability [6], leading to considerable health and socioeconomic burdens [7]. While low back pain is usually resolved within 6 weeks after onset, some patients still suffer from chronic low back pain [8], which accounts for approximately 40% of LBP patients [9].

Even with the high prevalence and high incidence of LBP, evidence regarding the causes of LBP is still inconclusive. Degenerative changes seen in spinal imaging do not explain the symptoms of LBP, as they are also seen in subjects without low back pain [10], and there is a weak correlation between symptoms of LBP and imaging [11]. An explicit pathological mechanism cannot be identified for 85% of LBP patients; hence, these patients are considered to have nonspecific LBP.

In nonspecific LBP, central sensitization (CS) is described by the International Association for the Study of Pain (IASP) as “Increased responsiveness of nociceptive neurons in the central nervous system to their normal or subthreshold afferent input” [12]. Abnormal pain processing in the central nervous system (CNS) rather than from actual damage and/or injury to bodily anatomic structures may lead to increased neuronal response and central sensitization (CS) [13], and this may manifest as mechanical hyperalgesia, allodynia, and/or referred pain, which are important in chronic pain syndromes [14]. While pain hypersensitivity measured by quantitative sensory testing might show no significant association with pain intensity or disability in patients with spinal pain [15], approximately one fourth of nonspecific LBP patients experience pain mainly originating from pain sensitization [16]. Moreover, a recent study revealed the difference in pain sensitization measured by mechanical quantitative sensory testing at the lower back between nonspecific chronic patients with low back pain and healthy controls [17].

### Description of the intervention

Various nonoperative interventions are available for nonspecific low back pain treatment, in which lifestyle modification, education, superficial heat, and some nonpharmacological therapy, including exercise and cognitive behavioral therapy, are considered the first-line treatment, while other nonpharmacological therapy and pharmacological therapy, both oral and systemic, are used as second-line or adjunctive treatments [18]. There are several nonpharmacological therapies with promising outcomes for nonspecific low back pain, such as massage [19], acupuncture [20, 21], yoga [22], and interdisciplinary rehabilitation, including biopsychosocial rehabilitation [23, 24], combined chiropractic [25], therapeutic ultrasound [26], radiofrequency denervation [27], neuroreflexotherapy [28], extracorporeal shockwave therapy [29–31], laser therapy [32–34], and neuromodulation [35–37]. For pharmacological therapies, nonsteroidal anti-inflammatory drugs (NSAIDs) [38–40], muscle relaxants [41], intra-articular facet joint injection [42], and herbal medicine, including *C. frutescens* (Cayenne), *H. procumbens*, *S. alba*, *S. officinale L*., *S. chilensis*, and lavender essential oil, have shown significant benefits for nonspecific LBP [43]. Ultrasound-guided injection and fluoroscopic-guided injection with promising outcomes have also been reviewed [44]. However, there is still a lack of relevant research, and additional studies need to be performed in the future.

### Mechanisms through which nonoperative treatment may improve pain sensitization

Pain sensitization is an important pain mechanism in patients with low back pain that relates to increasing pain intensity and disease progression while decreasing quality of life [45, 46]. Clinically, pain sensitization can be indirectly measured by quantitative sensory testing (QST) [47, 48]. Recently, there have been an increasing number of intervention studies on patients with low back pain presenting positive results on pain sensitization outcomes measured by the QST, particularly for pain pressure threshold (PPT) [49–51].

Patients with low back pain have decreased mechanical reception [52], decreased endogenous pain inhibition [53], decreased proprioception [54], and increased hyperalgesia [55]. Patients with low back pain can be classified by latent class analysis into pain “sensitized” and “not sensitized,” which might be an important factor of nonoperative treatment effectiveness on pain sensitization. The mechanisms of improving pain sensitization are different between patients with low back pain with high generalized pain sensitivity and those with increased local pain sensitivity [51]. Moreover, nociceptive input inhibition is a promising mechanism by which nonoperative treatment can improve pain sensitization [50].

### Why it is important to do this review

There are systematic reviews on observation studies comparing pain sensitization in patients with low back pain and healthy controls [17] and the association of pain sensitization and low back pain outcomes [56]. However, there is no systematic review on how nonoperative treatments affect pain sensitization in patients with low back pain. The systematic literature search and the review methods followed the current edition of the *Cochrane Handbook for Systematic Reviews of Interventions* [57] and recommendations from the Cochrane Back and Neck group [3].

### Objective

The objective is to evaluate the effectiveness of nonoperative treatment for pain sensitization measured by quantitative sensory testing in patients with nonspecific low back pain through a systematic review and meta-analysis.

## Methods

We have submitted the systematic review with the International Prospective Register of Systematic Reviews (PROSPERO) on March 20, 2021, and have followed the Preferred Reporting Items for Systematic Review and Meta-Analysis Protocols (PRISMA-P) 2015 statement [58]. We used the PRISMA-P 2015 checklist to establish the quality of the protocol (see Additional file 1).

### Criteria for considering studies for this review

#### Types of studies

We will include only original peer-reviewed articles of RCTs of nonoperative interventions. Nonrandomized intervention studies, observational studies, case series, case reports, review articles, letters to editors, commentaries, protocols, and guidelines will be excluded.

#### Type of participants

We will include RCTs of adult subjects defined as aged 18 years or older who have acute, subacute, or chronic nonspecific low back pain with or without sciatica. We will include RCTs that include participants of any gender and race/ethnicity, any duration of illness, and any previous treatment. We will exclude RCTs that include patients with specific low back pain caused by pathological entities such as infection, neoplasm, metastasis, osteoporosis, rheumatoid arthritis, cauda equina syndrome, or fractures. We will also exclude RCTs with pregnant or postpartum subjects.

#### Type of interventions

We will include RCTs with any type of nonoperative treatments (i.e., education, self-care, nonpharmacological therapy, pharmacological therapy, or interventional therapy) [18] administered by any route with or without the combination of other treatments as defined by the authors. The comparison intervention will be placebo, other nonoperative treatments, or surgical interventions.

#### Type of outcome measures

RCTs on this topic have various timing with respect to outcome measurements: immediate (postintervention to up to 7 days post-randomization), short-term (from the eighth day to less than 3 months post-randomization), intermediate-term (from 3 months to less than 6 months post-randomization), and long-term (6 months post-randomization or more). Therefore, we will examine each outcome described below at four time points: immediately, short-term, intermediate-term, and long-term. If there is more than one follow-up time point within each period, we will select the outcome measurement at the longest follow-up time point (i.e., if an RCT reports the outcomes at 1 week, 2 weeks, 1 month, 2 months, 3 months, 6 months, and 1 year, we will analyze the result at two months for the short-term outcome, three months for the intermediate-term outcome and one year for the long-term outcome).

#### Primary outcomes

- Mean change in static quantitative sensory testing, including but not limited to chemical, electrical, mechanical (pressure, punctate/brush, vibratory), and thermal (heat, cold), from the baseline.
- Mean change in dynamic quantitative sensory testing, including but not limited to temporal summation and conditioned pain modulation, from the baseline.
- Mean change in pressure pain threshold measured by pain algometry from baseline.

#### Secondary outcomes

- Proportion of patients who have intervention-related adverse events.

### Search methods for the identification of studies

#### Electronics searches

We will work with an information specialist to design an appropriate search strategy. The PubMed, Embase, Scopus, Web of Science, Cumulative Index to Nursing and Allied Health Literature (CINAHL), and Cochrane Central Register of Controlled Trials (CENTRAL) databases will be searched for peer-reviewed studies with no restrictions on publication date or language. In addition, the reference lists of included articles will be searched, as well as related citations from other journals via Google Scholar and Web of Science. Additional file 2 shows the detailed search strategies.

### Data collection and analysis

#### Selection of studies

We will use a systematic review management software (Covidence) to manage all citations identified from the systematic search [59]. After removing duplicate studies, two review authors will screen the titles and abstracts of potential studies independently. We will record each article as relevant or not relevant for full-text review. Two review authors will thoroughly read the full texts of relevant RCTs and use the inclusion and exclusion criteria to select RCTs for further data extraction. For each article excluded in the full-text screening, we will report the reason for exclusion. We will generate a study selection flow diagram to describe the workflow and identify the included RCTs. Discrepancies between two review authors in each stage of article screening will be resolved by consensus.

#### Data extraction and management

We will use Covidence to manage data extraction. We will prospectively design a data extraction form. The data extraction form will be pilot tested and refined. Two independent authors will extract the following data: (1) study information (authors, year of publication, study type, journal, contact, country, and funding), (2) characteristics of the participants (sample size, age, gender, ethnicity, comorbidities, current disease duration, presence of absence of sciatica), (3) intervention detail (type of intervention, duration of treatment, dosage, intervention compliance), (4) comparator detail (type of comparator, duration of treatment, dosage, comparator compliance), and (5) outcomes described under the “type of outcome measures” subheading (complete list of the names of all measured outcomes, unit of measurement, follow-up time point, missing data). All relevant text, tables, and figures will be examined for data extraction. Discrepancies between two independent authors will be resolved by consensus. We will contact the RCT authors to request incompletely reported data. If the RCT authors do not respond for 14 days, we will conduct analyses using available data. Multiple reports from the same study will be identified using authors’ names, funding sponsor, country, trial registration number, specific details of participants, and interventions. Then, we will collect data from each report and link the data together into one study.

#### Assessment of risk of bias in included studies

Two independent authors will assess the risk of bias in the included trials using the Cochrane Risk of Bias tool 2.0 for randomized control trial study [60]. We will assess each of the following domains:

- Bias arising from the randomization process.
- Bias due to deviations from intended intervention.
- Bias due to missing outcome data*.
- Bias in the measurement of the outcome*.
- Bias in the selection of the reported result.

*Domain will be assessed by the outcome.

We will assign each domain as having a low risk of bias, some concerns and a high risk of bias. We will contact the RCT authors if there is not enough information to assess. If the trial authors do not respond for 14 days, we will conduct an assessment using available data. We will resolve the disagreement through discussion. We will present our risk of bias assessment in the “Risk of bias” summary tables.

#### Assessment of reporting bias

We will search for trial protocols and trial registration. We will then compare the intended outcome measures and analyses specified in protocols and registration with those in the published articles. Reporting bias will be suspected when there is any change in primary outcomes, secondary outcomes, or statistical analysis plan.

#### Measure of treatment effect

For continuous outcomes, we will present the results as the mean difference with 95% confidence intervals (CIs), while we will present the results as the relative risk with 95% CIs for dichotomous outcomes.

#### Assessment of heterogeneity

We will assess statistical heterogeneity using the *I*^2^ and *X*^2^ statistics. We will categorize the level of heterogeneity for the *I*^2^ statistic as defined in chapter 9 of the Cochrane Handbook for Systematic Reviews of Interventions: 0 to 40% might not be important, 30 to 60% may represent moderate heterogeneity, 50 to 90% may represent substantial heterogeneity, and 75 to 100% may represent considerable heterogeneity [57]. For the *X*^2^ test, we will assess the included trials for statistical heterogeneity with a *P* value of less than 0.10 (statistically significant).

#### Data synthesis

We will provide qualitative analysis of trials and their results following standard 4.2 and conduct a qualitative synthesis, chapter 4 of Finding What Works in Health Care: Standards for Systematic Reviews [61]. If there is no considerable clinical, methodological, and statistical heterogeneity, we will use random effects meta-analysis by the DerSimonian and Laird method. The meta-analysis will be performed using Review Manager version 5.3 [62]. We will summarize the three primary outcome measures (mean change in static quantitative sensory testing from baseline; mean change in dynamic quantitative sensory testing from baseline; mean change in pressure pain threshold measured by pain algometry from baseline) at two specific time points, including short-term and intermediate-term time points along with adverse event outcome measures, in the “Summary of findings” table.

#### Quality of evidence

We will use the Grading of Recommendation Assessment, Development and Evaluation (GRADE) approach to assess the quality of evidence for the primary outcome (i.e., mean change in static quantitative sensory testing from the baseline; mean change in dynamic quantitative sensory testing from the baseline; mean change in pressure pain threshold measure by pain algometry). We will use the five GRADE considerations, including risk of bias, imprecision, inconsistency, indirectness, and publication bias. We will then classify each outcome as follows [63]:

- High quality was defined as “we are very confident that the true effect lies close to that of the estimate of the effect.”
- Moderate quality is defined as “we are moderately confident in the effect estimate: the true effect is likely to be close to estimate of the effect, but there is a possibility that it is substantially different.”
- Low quality, defined as “our confidence in the effect estimate is limited: the true effect may be substantially different from the estimate of the effect.”
- Very low quality defined as “we have very little confidence in the effect estimate: the true effect is likely to be substantially different from the estimate of effect.

#### Subgroup analysis and investigation of heterogeneity

We will undertake a subgroup analysis by type of intervention, duration of intervention (immediate, short-term, intermediate-term, and long-term), type of comparator, presence or absence of sciatica, and disease duration (acute and chronic).

#### Sensitivity analysis

We will exclude RCTs with a high risk of overall bias to assess the robustness of the results. We will conduct additional sensitivity analyses to determine the impact of any post hoc decisions, if any, during the review process.

## Supplementary Information

**Additional file 1.** PRISMA-P 2015 Checklist.

**Additional file 2.** Details of search strategies for each database.

## Data Availability

Data will be available as supplementary files.

## Abbreviations

CENTRAL: Cochrane Central Register of Controlled Trials
CIs: Confidence intervals
GRADE: Grading of Recommendation Assessment, Development and Evaluation
LBP: Low back pain
PRISMA-P: Preferred Reporting Items for Systematic review and Meta-analysis Protocols
QST: Quantitative sensory testing
RCTs: Randomized controlled trials
US: United States
