# Supplementary material for: Nonoperative treatment for pain sensitization in patients with low back pain: protocol for a systematic review
Source: Syst Rev. 2022 Apr 4;11:59. doi: 10.1186/s13643-022-01927-2 (PMC8981789; doi:10.1186/s13643-022-01927-2)
Supplement: Supplementary file 2 — Additional file 2. Details of search strategies for each database. [file 13643_2022_1927_MOESM2_ESM.docx]

**Additional file 2: Details of search strategies for each database**

**Database: PubMed search strategy**

#1 “back pain”[MeSH Terms]
#2 “back pain”[tiab]
#3 backache[tiab]
#4 dorsalgia[tiab]
#5 “back disorder”[tiab]
#6 “back disorders”[tiab]
#7 ((lumb*[tiab] OR back[tiab]) AND (pain[tiab] OR disorder*[tiab]))
#8 coccyx[tiab]
#9 coccydynia[tiab]
#10 sciatica[MeSH Terms]
#11 “sciatic neuropathy”[MeSH Terms]
#12 sciatic*[tiab]
#13 “low back pain”[MeSH Terms]
#14 spondylosis[tiab]
#15 lumbago[tiab]
#16 (#1 OR #2 OR #3 OR #4 OR #5 OR #6 OR #7 OR #8 OR #9 OR #10 OR #11 OR #12 OR #13
 OR #14 OR #15)
#17 “quantitative sensory”[tiab]
#18 qst[tiab]
#19 “pain threshold”[MeSH Terms]
#20 “sensory thresholds”[MeSH Terms]
#21 “pain threshold”[tiab]
#22 “pain thresholds”[tiab]
#23 (pain[tiab] AND (threshold*[tiab] OR sensitiz*[tiab]))
#24 algomet*[tiab]
#25 “temporal summation”[tiab]
#26 “pain modulation”[tiab]
#27 “pain sensitization”[tiab]
#28 (#17 OR #18 OR #19 OR #20 OR #21 OR #22 OR #23 OR #24 OR #25 OR #26 OR #27)
#29 “randomized controlled trial”[pt]
#30 “controlled clinical trial”[pt]
#31 “pragmatic clinical trial”[pt]
#32 random*[tiab]
#33 placebo[tiab]
#34 "drug therapy"[sh]
#35 trial[tiab]
#36 groups[tiab]
#37 (#28 OR #29 OR #30 OR #31 OR #32 OR #33 OR #34 OR #35 OR #36)
#38 (#16 AND #28)
#39 (#37 AND #38)
#40 (animals[MeSH Terms] NOT humans[MeSH Terms])
#41 (#39 NOT #40)

**Database: Embase search strategy**

#1 'backache'/exp
#2 'back pain':ti,ab
#3 'backache':ti,ab
#4 'dorsalgia':ti,ab
#5 'back disorder':ti,ab
#6 'back disorders':ti,ab
#7 (('lumb*':ti,ab OR 'back':ti,ab) AND ('pain':ti,ab OR 'disorder*':ti,ab))
#8 'coccyx':ti,ab
#9 'coccydynia':ti,ab
#10 'sciatica'/exp
#11 'sciatic*':ti,ab
#12 'low back pain'/exp
#13 'sciatic neuropathy'/exp
#14 'ischialgia'/exp
#15 'spondylosis':ti,ab
#16 'lumbago':ti,ab
#17 (#1 OR #2 OR #3 OR #4 OR #5 OR #6 OR #7 OR #8 OR #9 OR #10 OR #11 OR #12 OR #13
 OR #14 OR #15 OR #16)
#18 'quantitative sensory':ti,ab
#19 'qst':ti,ab
#20 'pain threshold'/exp
#21 'perceptive threshold'/exp
#22 'pain threshold':ti,ab
#23 'pain thresholds':ti,ab
#24 ('pain':ti,ab AND ('threshold*':ti,ab OR 'sensitiz*':ti,ab))
#25 'algomet*':ti,ab
#26 'temporal summation':ti,ab
#27 'pain modulation':ti,ab
#28 'pain sensitization':ti,ab
#29 (#18 OR #19 OR #20 OR #21 OR #22 OR #23 OR#24 OR #25 OR #26 OR #27 OR #28)
#30 'randomized controlled trial'/exp
#31 'controlled clinical trial'/exp
#32 'controlled study'/exp
#33 'double blind procedure'/exp
#34 'single blind procedure'/exp
#35 'crossover procedure'/exp
#36 'placebo'/exp
#37 'randomization'/exp
#38 'random*':ti,ab
#39 'placebo?':ti,ab
#40 'allocat*':ti,ab
#41 'assign*':ti,ab
#42 'blind*':ti,ab
#43 'cross-over':ti,ab OR 'crossover':ti,ab
#44 'compare':ti,ab OR 'compared':ti,ab OR 'comparing':ti,ab OR 'comparison':ti,ab OR
 'comparative':ti,ab
#45 ('controlled':ti,ab AND ('study':ti,ab OR 'design':ti,ab))
#46 (('singl*':ti,ab OR 'doubl*':ti,ab OR 'trebl*':ti,ab OR 'tripl*':ti,ab) AND 'mask*':ti,ab)
#47 'trial':ti,ab
#48 (#29 OR #30 OR #31 OR #32 OR #33 OR #34 OR #35 OR #36 OR #37 OR #38 OR #39 OR #40
 OR #41 OR #42 OR #43 OR #44 OR #45 OR #46 OR #47)
#49 (#17 AND #29)
#50 (#48 AND #49)
#51 ([animals]/lim NOT [humans]/lim)
#52 (#50 NOT #51)

**Database: CENTRAL search strategy**

#1 MeSH descriptor: [Back Pain] explode all trees

#2 “back pain”:ti,ab,kw
#3 backache:ti,ab,kw
#4 dorsalgia:ti,ab,kw
#5 “back disorder”:ti,ab,kw
#6 “back disorders”:ti,ab,kw
#7 ((lumb*:ti,ab,kw OR back:ti,ab,kw) NEAR/3 (pain:ti,ab,kw OR disorder*:ti,ab,kw))
#8 coccyx:ti,ab,kw
#9 coccydynia:ti,ab,kw
#10 MeSH descriptor: [Sciatica] explode all trees
#11 MeSH descriptor: [Sciatic Neuropathy] explode all trees
#12 sciatic*:ti,ab,kw
#13 MeSH descriptor: [Low Back Pain] explode all trees
#14 spondylosis:ti,ab,kw
#15 lumbago:ti,ab,kw
#16 discitis:ti,ab,kw
#17 (disc:ti,ab,kw NEAR herniat*:ti,ab,kw)
#18 “spinal fusion”:ti,ab,kw
#19 (facet:ti,ab,kw NEAR joint*:ti,ab,kw)
#20 MeSH descriptor: [Intervertebral Disc] explode all trees
#21 arachnoiditis:ti,ab,kw
#22 (failed:ti,ab,kw NEAR back:ti,ab,kw)
#23 (lumbar:ti,ab,kw NEAR vertebra*:ti,ab,kw)
#24 ((slipped:ti,ab,kw OR degenerat*:ti,ab,kw OR displace*:ti,ab,kw OR prolap*:ti,ab,kw)
 NEAR (disc*:ti,ab,kw OR disk*:ti,ab,kw))
#25 (stenosis:ti,ab,kw NEAR (spine:ti,ab,kw OR root:ti,ab,kw OR spinal:ti,ab,kw))
#26 (#1 OR #2 OR #3 OR #4 OR #5 OR #6 OR #7 OR #8 OR #9 OR #10 OR #11 OR #12 OR #13
 OR #14 OR #15 OR #16 OR #17 OR #18 OR #19 OR #20 OR #21 OR #22 OR #23 OR #24
 OR #25)
#27 “quantitative sensory”:ti,ab,kw
#28 qst:ti,ab,kw
#29 MeSH descriptor: [Pain Threshold] explode all trees
#30 MeSH descriptor: [Sensory Thresholds] explode all trees
#31 “pain threshold”:ti,ab,kw
#32 “pain thresholds”:ti,ab,kw
#33 (pain:ti,ab,kw AND (threshold*:ti,ab,kw OR sensitiz*:ti,ab,kw)
#34 algomet*:ti,ab,kw
#35 “temporal summation”:ti,ab,kw
#36 “pain modulation”:ti,ab,kw
#37 “pain sensitization”:ti,ab,kw
#38 (#27 OR #28 OR #29 OR #30 OR #31 OR #32 OR #33 OR #34 OR #35 OR #36 OR #37)
#39 (#26 AND #38)

**Database: CINAHL search strategy**

S51 S50 AND S40 AND S24
S50 S49 OR S48 OR S47 OR S46 OR S45 OR S44 OR S43 OR S42 OR S41
S49 pain sensitization
S48 pain modulation
S47 temporal summation
S46 algomet*
S45 (pain AND (threshold* OR sensitiz*))
S44 pain thresholds
S43 pain threshold
S42 qst
S41 quantitative sensory
S40 S39 OR S38 OR S37 OR S36 OR S35 OR S34 OR S33 OR S32 OR S31 OR S30 OR S29 OR
 S28 OR S27 OR S26 OR S25
S39 lumbago
S38 spondylosis
S37 (MH “Low Back Pain”)
S36 sciatic*
S35 (MH “Sciatica”)
S34 coccydynia
S33 coccyx
S32 (MH “Coccyx”)
S31 ((lumb* OR back) AND (pain OR disorder*))
S30 back disorders
S29 back disorder
S28 dorsalgia
S27 backache
S26 back pain
S25 (MH “Back Pain+”)
S24 S22 NOT S23
S23 (MH “Animals+”)
S22 S21 OR S20 OR S19 OR S18 OR S17 OR S16 OR S15 OR S14 OR S13 OR S12 OR S11 OR
 S10 OR S9 OR S8 OR S7 OR S6 OR S5 OR S4 OR S3 OR S2 OR S1
S21 volunteer*
S20 control*
S19 followup stud*
S18 follow-up stud*
S17 (MH “Evaluation Research+”)
S16 prospectiv*
S15 (MH “Prospective Studies+”)
S14 (MH “Comparative Studies”)
S13 latin square
S12 (MH “Study Design+”)
S11 random*
S10 (MH “Random Sample+”)
S9 placebo*
S8 (MH “Placebos”)
S7 (MH “Placebo Effect”)
S6 single-blind
S5 triple-blind
S4 double-blind
S3 clinical W3 trial
S2 “randomi?ed controlled trial*”
S1 (MH “Clinical Trials+”)

**Database: SCOPUS search strategy**

#1 TITLE-ABS-KEY(“back pain” OR backache OR dorsalgia OR “back disorder” OR “back
 disorders” OR ((lumb* OR back) W/3 (pain OR disorder*)) OR coccyx OR coccydynia OR
 sciatic* OR spondylosis OR lumbago OR discitis OR (disc W/6 herniat*) OR “spinal fusion” OR
 (facet W/6 joint*) OR arachnoiditis OR (failed W/6 back) OR (lumbar W/6 vertebra*) OR
 ((slipped OR degenerat* OR displace* OR prolap*) W/6 (disc* OR disk*)) OR (stenosis W/6
 (spine OR root OR spinal)))

#2 TITLE-ABS-KEY(“quantitative sensory” OR qst OR “pain threshold” OR “pain thresholds” OR
(pain AND (threshold* OR sensitiz*)) OR algomet* OR “temporal summation” OR “pain modulation” OR “pain sensitization”)

#3 TITLE-ABS-KEY(trial OR trials OR random* OR placebo* OR sham OR control* OR ((singl*
 OR doubl* OR trebl* OR tripl*) W/7 mask*) OR allocat* OR assign* OR blind* OR group* OR
 compare OR compared OR comparing OR comparison OR comparative OR cross-over OR
 crossover)

#4 (#1 AND #2)

#5 (#3 AND #4)

#6 ALL(animals AND NOT humans)

#7 (#5 AND NOT #6)

**Database: Web of Science search strategy**

#1 TS=(“back pain” OR backache OR dorsalgia OR “back disorder” OR “back disorders” OR
 ((lumb* OR back) near/3 (pain OR disorder*)) OR coccyx OR coccydynia OR sciatic* OR
 spondylosis OR lumbago OR discitis OR (disc near/6 herniat*) OR “spinal fusion” OR (facet
 near/6 joint*) OR arachnoiditis OR (failed near/6 back) OR (lumbar near/6 vertebra*) OR
 ((slipped OR degenerat* OR displace* OR prolap*) near/6 (disc* OR disk*)) OR (stenosis near/6
 (spine OR root OR spinal)))

#2 TS=(“quantitative sensory” OR qst OR “pain threshold” OR “pain thresholds” OR (pain AND
(threshold* OR sensitiz*)) OR algomet* OR “temporal summation” OR “pain modulation” OR “pain sensitization”)

#3 TS=(trial OR trials OR random* OR placebo* OR sham OR control* OR ((singl* OR doubl* OR
 trebl* OR tripl*) near/7 mask*) OR allocat* OR assign* OR blind* OR group* OR compare OR
 compared OR comparing OR comparison OR comparative OR cross-over OR crossover)

#4 (#1 AND #2)

#5 (#3 AND #4)

#6 ALL=(animal NOT human)

#7 (#5 NOT #6)
